# Shorter incubation period is associated with severe disease progression in patients with COVID-19

**DOI:** 10.1080/21505594.2020.1836894

**Published:** 2020-10-27

**Authors:** Changxiang Lai, Rentao Yu, Mingbo Wang, Wenjie Xian, Xin Zhao, Qiyuan Tang, Ruikun Chen, Xuan Zhou, Xuan Li, Zhiyu Li, Zhiwei Li, Guohong Deng, Fang Wang

**Affiliations:** a Department of Liver Diseases, The Third People’s Hospital of Shenzhen, National Clinical Research Center for Infectious Disease, the Second Affiliated Hospital of Southern University of Science and Technology, Shenzhen, Guangdong, China; b Department of Infectious Diseases, Southwest Hospital, Third Military Medical University (Army Medical University), Chongqing, China; c Department of Infectious Diseases, The General Hospital of Western Theater Command, Chengdu, Sichuan, China; d Department of Hepatobiliary Surgery, The Third People’s Hospital of Shenzhen, National Clinical Research Center for Infectious Disease, the Second Affiliated Hospital of Southern University of Science and Technology, Shenzhen, Guangdong, China

**Keywords:** incubation period, COVID-19, SARS-CoV-2, disease aggravation, CT scores

## Abstract

The diagnosed COVID-19 cases revealed that the incubation periods (IP) varied a lot among patients. However, few studies had emphasized on the different clinical features and prognosis of patients with different IP. A total of 330 patients with laboratory-confirmed COVID-19 were enrolled and classified into immediate onset group(IP<3 days, I group, 57 cases) and late onset group(IP>10 days, L group, 75 cases) based on IP. The difference of clinical characteristics and prognosis of the two groups were compared. There were more patients with fever in I group than in L group(P = 0.003), and counts of all the total lymphocytes, total T lymphocytes, CD4 + and CD8 + T lymphocytes were significantly different between the two groups(all P < 0.01). Besides, patients in L group had more GGOs in CT scan than I group and there were more patients in I group receiving antibiotic treatment than in L group(P < 0.001). For disease aggravation, the median CT scores were comparable between the two groups, but individually, there were more patients with increased CT score during hospitalization in I group than in L group. The aggravation incidence of CT presentation was 21.1% in I group, significantly higher than L group(8.0%, P = 0.042). Multivariable COX models suggested that IP was the only independent factors for CT aggravation. Conclusively, patients with different IP were different in clinical symptoms, laboratory tests, and CT presentations. Shorter IP was associated with the aggravation of lung involvement in CT scan.

## Introduction

Starting in December 2019, several cases of unidentified viral pneumonia with similar clinical manifestations were diagnosed in Wuhan, and then, evidence of person-to-person transmission were reported [1]. The next-generation sequencing of respiratory samples revealed that a novel coronavirus, subsequently named SARS-CoV-2, was the infectious agent, and this new disease was then named COVID-19 by World Health Organization (WHO). Cases of COVID-19 have been increasing rapidly worldwide, with more than 400,000 cases confirmed (by 10^th^ May), and the total number of cases and deaths outside China has exceeded the total number of cases and deaths in China since mid-March. On March 11, 2020, the WHO declared COVID-19 pandemic.

Consistent with severe acute respiratory syndrome (SARS) [2]and Middle Eastern Respiratory Syndrome (MERS) [3], the symptomatic manifestations of SARS-CoV-2-induced pneumonia are similar. Fever, cough, and dyspnea are the most common clinical manifestations, while the majority of chest CTs show a ground glass opacity(GGO) [4,5]. Statistically, it is reported that 20% to 30% of patients would develop into severe condition requiring mechanical ventilation therapy, while up to 10% of patients would subsequently die [6]. In contrast, there are still many infected patients without any clinical symptoms or radiological abnormalities [7]. Consequently, the current prevention and control situation remains critical.

With increasing more studies on COVID-19, the epidemiological features, clinical symptoms, imaging characteristics, and therapeutic precautions of COVID-19 are now well understood. Although most patients with COVID-19 present with lung opacity, severe complications are only observed in a small subgroup of patients. It has been proven that immune system plays a vital role in COVID-19 prognosis. The hyperinflammatory response to SARS-CoV-2 is thought to be a major cause of disease severity and death in patients with COVID-19 and is associated with high levels of serum cytokines (cytokine storm) [8]. In the aspect of clinical symptoms, the onset time of symptom was different among different people. Some patients started to fever, cough or have headache immediately after exposure to patients or carriers, while some started after more than 10 days, given that the SARS-CoV-2 is susceptible to all populations. However, research on the prognosis of patients with different incubation periods had barely been reported.

In this study, the clinical and radiological characteristics of COVID-19 patients were retrospectively analyzed to compare the differences between patients with different incubation periods, and to further compare the aggravation rate between the two groups.

## Patients and methods

### Study population

This retrospective study was conducted in the Second Hospital Affiliated to Southern University of Science and Technology in Shenzhen, Guangdong. A total of 330 patients with laboratory-confirmed COVID-19 admitted to the hospital between Jan. 11^th^ and Feb. 10^th^, 2020 were enrolled, and followed up until Feb. 23^rd^, 2020. All the enrolled patients were diagnosed and COVID-19 was confirmed if at least two consecutive positive results by real-time polymerase chain reaction (PCR) assay for SARS-CoV-2 or a genetic sequence that matches SARS-CoV-2 were obtained [9]. Clinical types, classified as minimal, common, severe, and critical, were determined by the Diagnosis and Treatment Program of Pneumonia of New Coronavirus Infection (Trial Seventh Edition) recommended by China’s National Health Commission [10].

The study protocols were approved by the Ethic Committee of The Third People’s Hospital of Shenzhen. As a retrospective study, the informed contents from all enrolled patients were waived under the authorization of the Ethic Committee.

### Data collection

Epidemiological, clinical, laboratory, therapeutic, CT, and outcome data were collected from patients’ medical records, and verified by two independent doctors or radiologists. Incubation period was defined as the time interval between the exposure and the onset of symptoms. The exposure time (travel from Hubei or high burden area; exposure to people from Hubei or high burden area) was determined by inquiring medical history and travel track information provided by big data and artificial intelligence (from smartphones, mobile payment, closed-circuit television, high-speed rail or airplane, etc.), and was finally decided by doctors. The time of symptom onset was provided by patients. Patients with an incubation period of less than 3 days were classified into immediate onset group (I group, 57 cases), while patients with an incubation time of more than 10 days were classified into late onset group (L group, 75 cases). The remaining patients were excluded from this analysis to get a clear classification of immediate onset and late onset time.

Disease aggravation was defined by two aspects: (1) clinical aggravation:evaluation based on clinical types during hospitalization or intensive care intervention; (2) image aggravation: the aggravation of CT presentation based on CT scores.

### CT analysis

The CT characteristics were described using standard nomenclature recommended by the Fleischner Society glossary and peer-reviewed literature, defined as GGO, crazy-paving pattern, and consolidation [11]. Besides, the presence of nodules, pleural effusion, thoracic lymphadenopathy (defined as lymph node size of ≥10 mm in short-axis dimension), lung cavitation, emphysema, and fibrosis was also recorded [12]. A semi-quantitative scoring system was used to estimate the opacities involvement of all abnormalities, as described by previous studies [13,14]. The total CT score ranged from 0 (no involvement) to 25 (maximum involvement).

The distribution of lung abnormalities was also recorded as subpleural (involving mainly the peripheral one-third of the lung) and random (without predilection for subpleural or central regions) [5].

### Statistical analysis

The data were analyzed using R software (version 3.6.1, www.r-project.org). Quantitative variables were represented as median with interquartile range, and the comparisons of variables between two groups were performed using Student t-test if data were homogeneous of variance and normally distributed by Kolmogorov–Smirnov test, or else using Mann–Whitney test. Categorical variables were represented as number with percentage and the comparison was performed using χ2 test. Kaplan–Meier curve was depicted to determine the incidence of disease aggravation and the Log-rank P was calculated to compared the difference of two groups. Adjusted and multivariable COX hazard analysis, using different models including different variables, were applied to determine the independent factors for disease aggravation. Two-tailed P value less than 0.05 was regarded as statistically significant.

## Results

### Demographic characteristics of enrolled patients

The demographical and epidemiological characteristics of the two groups and total enrolled patients are listed in Table 1. The median age of enrolled patients was 47.0 (33.0–60.0) years, but patients in I group were significantly older than that in L group (52.0 vs 36.0 yrs, P < 0.001). For exposure history, 37.6% diagnosed patients came from Hubei, while 57.3% local patients had contact history. Interestingly, there were more patients from Hubei in I group than L group (47.4% vs 13.3%, P < 0.001). Besides, the patients in I group stayed longer in hospital than L group (16.0 vs 12.0 days, P < 0.001). A total of 77 patients (23.3%) had chronic disease, and there was no significant difference between I and L group. Apart from SARS-CoV-2, 6 patients were coinfected with Flu A/B virus. Around 70% enrolled patients were classified into common type; However, patients in I group were more severe than L group (P < 0.001).

**Table 1.** Demographical characteristics of enrolled patients categorized by onset time

	Immediate onset (N = 57)	Late onset (N = 75)	P	Total (N = 330)
**Age, years**	52.0(32.0–60.5)	36.0(12.0–53.0)	<0.001	47.0(33.0–60.0)
**Gender, female**	35(61.4%)	39(52.0%)	0.281	169(52.2%)
**Onset of exposure to symptom, days**	2.0(1.0–3.0)	14.0(12.0–18.0)	<0.001	7.0(4.0–12.0)
**Length of stay, days**	16.0(14.0–20.0)	12.0(8.0–15.0)	<0.001	15.0(13.0–18.0)
**Preexisting** **conditions**				
Any	10(17.5%)	9(12.0%)	0.369	77(23.3%)
Diabetes	2(3.5%)	1(1.3%)	0.393	18(5.5%)
Hypertension	5(8.8%)	6(8.0%)	0.874	38(11.5%)
Chronic liver disease	2(3.5%)	0	0.102	8(2.4%)
COPD	2(3.5%)	1(1.3%)	0.406	11(3.3%)
Heart disease	1(1.8%)	0	0.250	15(4.5%)
Cancer	1(1.8%)	1(1.3%)	0.844	3(0.9%)
Cerebrovascular disease	1(1.8%)	0	0.250	2(0.6%)
**Coinfected with other virus**	2(3.5%)	3(4.0%)	0.884	6(1.8%)
**Clinical type on admission**			<0.001	
Mild	6(10.5%)	24(32.0%)		74(22.4%)
Common	47(82.5%)	48(64.0%)		227(68.8%)
Severe	4(7.0%)	3(4.0%)		25(7.6%)
Critical	0	0		4(1.2%)

### Comparison of clinical symptom, laboratory tests, and CT characteristics

Clinical symptoms and laboratory tests were compared between the two groups in Table 2. There were no difference in body temperature between I group and L group (P = 0.125), but there were more patients with fever in I group than in L group (47 vs 44, P = 0.003). For other symptoms, there were no significant difference between the two groups, except sputum production (P = 0.020). Patients in I group varied a lot with L group for results of blood tests. Generally, white blood cell counts in I group were significantly lower than L group (4.36 vs 4.79 × 109/L, P = 0.032). Specifically, counts of all the total lymphocytes, total T lymphocytes, CD4 + T lymphocytes and CD4 + T lymphocytes were significantly different between the two groups (all P < 0.01). Patients in L group had more lymphocytes in blood than I group. Interestingly, level of procalcitonin in I group was significantly higher than L group (P < 0.001), but the median levels were both under normal limit.

**Table 2.** Clinical symptoms and laboratory tests of enrolled patients categorized by onset time

	Immediate onset (N = 57)	Late onset (N = 75)	P	Total (N = 330)
**Body temperature, °C**	37.0(36.6–37.5)	36.7(36.5–37.2)	0.125	37.0(36.6–37.5)
<37.3	38(66.7%)	57(76.0%)		212(64.2%)
37.3–38	16(28.1%)	10(13.3%)		86(26.1%)
38.1–39	3(5.3%)	8(10.7%)		31(9.4%)
≥39	0	0		1(0.3%)
**Symptoms on admission**				
Fever	47(82.5%)	44(58.7%)	0.003	254(77.0%)
Cough	31(54.4%)	28(37.3%)	0.051	166(50.3%)
Sputum production	16(28.1%)	9(12.0%)	0.020	167(50.6%)
Shortness of breath	2(3.5%)	1(1.3%)	0.406	14(4.2%)
Fatigue	9(15.8%)	6(8.0%)	0.162	74(22.4%)
Loss of appetite	2(3.5%)	8(10.7%)	0.124	45(13.6%)
Nausea	0	2(2.7%)	0.214	10(3.0%)
Headache	6(10.5%)	2(2.7%)	0.061	27(8.2%)
Diarrhea	5(8.8%)	5(6.7%)	0.651	25(7.6%)
Sore throat	0	1(1.3%)	0.382	5(1.5%)
Nasal obstruction	0	1(1.3%)	0.382	2(0.6%)
**Blood tests**				
WBC, ×109/L	4.36(3.11–5.57)	4.79(3.80–5.84)	0.032	4.60(3.58–5.72)
Neutrophils, ×109/L	2.59(1.64–3.69)	2.42(1.68–3.31)	0.883	2.58(1.89–3.48)
Lymphocytes, ×109/L	1.17(0.99–1.38)	1.56(1.12–2.29)	<0.001	1.27(0.98–1.73)
Platelets, ×109/L	166.0(138.5–208.5)	191.0(160.0–253.0)	0.007	180.0(143.0–224.0)
Hemoglobin, g/L	135.0(127.5–144.5)	137.0(123.0–145.0)	0.746	136.0(126.8–146.0)
T lymphocyte	744.0(481.0–1165.0)	1198.0(882.3–1621.0)	0.006	970.5(627.3–1319.0)
CD4 + T lymphocyte	446.0(244.5–634.0)	625.0(447.0–911.5)	0.003	519.0(344.3–714.8)
CD8 + T lymphocyte	326.0(187.0–511.0)	442.5(325.0–655.0)	0.002	347.5(207.5–506.5)
**Blood chemistry**				
TBIL, umol/L	10.3(7.8–13.6)	9.2(6.7–14.6)	0.294	9.8(7.6–14.6)
ALT, U/L	20.0(12.5–27.5)	19.0(15.0–29.0)	0.883	20.0(15.0–31.0)
AST, U/L	24.0(19.5–30.5)	28.0(21.0–38.0)	0.073	27.0(21.0–36.5)
BUN, mmol/L	4.1(3.2–5.1)	3.8(3.1–4.7)	0.377	3.9(3.2–4.9)
Cr, umol/L	64.0(48.5–78.0)	54.0(42.0–71.6)	0.024	62.5(50.0–75.4)
LDH, U/L	197.0(157.0–307.0)	238.0(186.5–480.0)	0.011	233.0(176.0–400.0)
cTnI, ug/L	0.012(0.006–0.012)	0.012(0.007–0.012)	0.202	0.012(0.006–0.012)
Creatine kinase, U/L	78.0(51.5–123.0)	72.0(49.3–92.9)	0.628	71.0(51.0–103.0)
**Coagulation function**				
PT, s	11.9(11.1–12.5)	11.8(11.3–12.6)	0.514	11.9(11.3–12.5)
D-mer, s	0.39(0.29–0.63)	0.31(0.25–0.54)	0.067	0.37(0.26–0.55)
**Infection-related biomarkers**				
Procalcitonin, ng/mL	0.046(0.031–0.076)	0.031(0.021–0.050)	<0.001	0.042(0.026–0.065)
C reactive protein, mg/L	9.9(4.3–26.4)	5.3(1.9–20.6)	0.054	10.3(3.9–27.3)
IL6, pg/ml	8.59(5.08–17.25)	4.82(3.12–15.96)	0.069	10.91(4.16–19.57)

Baseline CT characteristics were also analyzed and compared in Table 3. In general, COVID-19 patients had similar CT features, but there were still some differences. Among all the patients undergoing CT tests, patients were equally distributed for lobes involvement, with about 80% patients involving both lateral lungs. For opacity characteristics, about 70% opacities distributed peripherally and the mixture of GGO and consolidation could be regularly seen in COVID-19 patients. But relatively, patients in L group were prone to have GGOs in CT scan. Besides, lung cavitation and emphysema were found only in I group, but not in L group.

**Table 3.** CT characteristics of enrolled patients categorized by onset time

	Immediate onset (N = 48)	Late onset (N = 61)	P	Total (N = 296)
**CT scores**	5.0(2.0–9.0)	7.0(3.0–11.0)	0.198	6.0(2.0–11.0)
**Number of lobes involved**			0.637	
0	5(10.4%)	4(6.6%)		25(8.4%)
1 lobe	7(14.6%)	6(9.8%)		31(10.5%)
2 lobes	8(16.7%)	8(13.1%)		46(15.5%)
3 lobes	9(18.8%)	9(14.8%)		50(16.9%)
4 lobes	7(14.6%)	16(26.2%)		44(14.9%)
5 lobes	12(25.0%)	18(29.5%)		84(28.4%)
**Bilateral Involvement**	39(81.3%)	54(88.5%)	0.287	236(79.7%)
**Opacity distribution**			0.461	
Peripheral	34(70.8%)	47(77.0%)		203 (68.6%)
Central	14(29.2%)	14(23.0%)		93(31.4%)
**Opacity patterns**				
Pure GGO	19(39.6%)	43(70.5%)	0.001	203(68.6%)
Consolidation	33(68.8%)	51(83.6%)	0.067	210(70.9%)
“Crazy-Paving” Pattern	17(35.4%)	17(27.9%)	0.398	101(34.1%)
Nodules	0	0	NA	1(0.3%)
Linear opacities	19(39.6%)	29(47.5%)	0.406	139(47.0%)
Lung cavitation	3(6.3%)	0	0.048	10(3.4%)
**Other findings**				
Lymphadenopathy	0	0	NA	0
Pleural effusion	0	0	NA	0
Emphysema	2(4.2%)	0	0.108	2(0.7%)
Fibrosis	5(10.4%)	8(13.1%)	0.666	39(13.2%)

### Treatment and prognosis

Therapeutic procedures were applied based on the Diagnosis and Treatment Program of Pneumonia of New Coronavirus Infection (Trial 7^th^ edition), as shown in Table 4. It should be noted that there were more patients in I group receiving antibiotic treatment than in L group (P < 0.001), suggesting the higher rate of bacteria coinfection in I group. There were no significant difference in other treatments.

**Table 4.** Treatment and prognosis during hospitalization of enrolled patients categorized by onset time

	Immediate onset (N = 57)	Late onset (N = 75)	P	Total (N = 330)
**Treatment**				
Anti-coronavirus treatment	57(100%)	75(100%)	1	330(100%)
Glucocorticoids	15(26.3%)	14(18.7%)	0.293	89(27.0%)
Antibiotic treatment	22(38.6%)	8(10.7%)	<0.001	98(29.7%)
Immunoglobulin	15(26.3%)	11(14.7%)	0.096	80(24.2%)
Regulating gut microbiome	30(52.6%)	40(53.3%)	0.936	179(54.2%)
Mechanical ventilation	4(7.0%)	3(4.0%)	0.443	39(11.8%)
ECMO	0	0	NA	0
CRRT	0	0	NA	2(0.6%)
**Aggravation during hospitalization**				
From mild/common type to severe type	10(17.5%)	9(12.0%)	0.369	62(18.8%)
From severe type to critical type	1(1.8%)	0	0.250	13(3.9%)
Admission to ICU	2(3.5%)	1(1.3%)	0.406	22(6.7%)
Any	10(17.5%)	9(12.0%)	0.369	70(21.2%)

**Figure 1.**
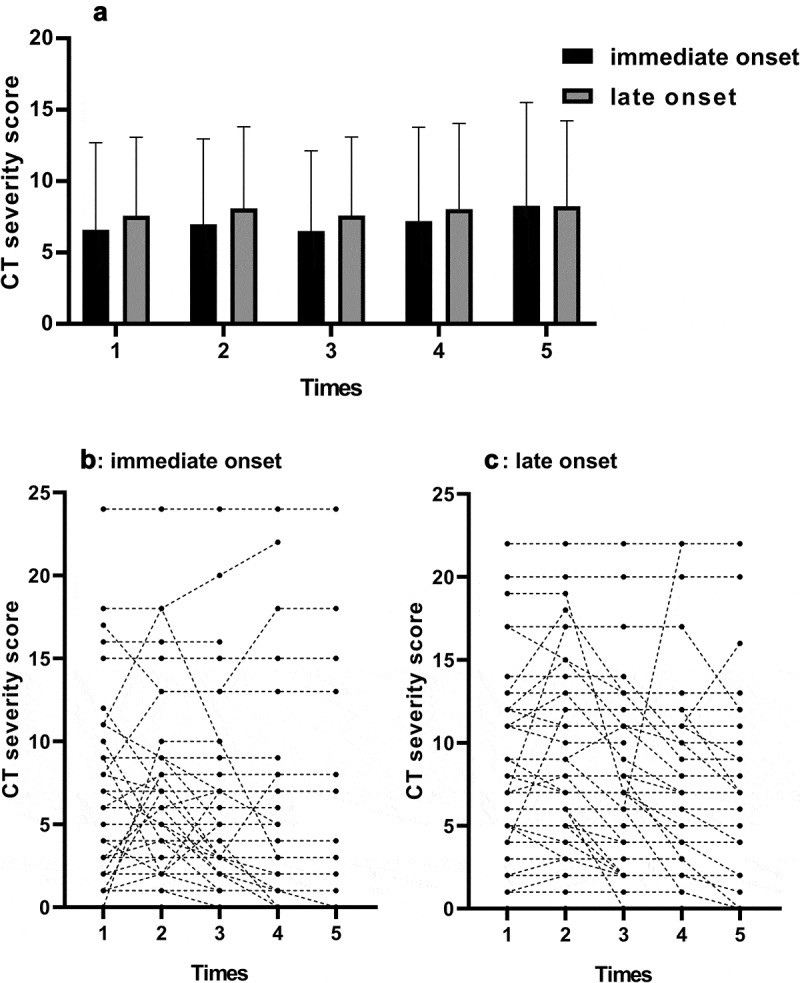
Change trend of CT scores. (a) the overall change of the two group (median with 95% confidence interval). (b) the individual change in I group (c). the individual change in L group. This figure represented different CT severity score of each CT scan during hospitalization. In (b) and (c), each line and dot represented a single patient’s CT score in consecutive times. As the figure showed, there were more patients in I group with an elevated severity scores than in I group during first two examinations, which means more patients exacerbated in CT scan in the beginning

Most patients recovered during hospitalization, while some patients aggravated based on clinical types and intensive care unit (ICU) admission, covering 21.2%. However, there were no significant difference between I and L group. The changes of CT presentations also recorded and shown in Figure 1. The median CT scores were comparable between the two groups, but individually, things were different. As Figure 1(b,c) showed, there were more patients with increased CT score during hospitalization in I group than in L group.

### Factors that influence disease aggravation

**Figure 2.**
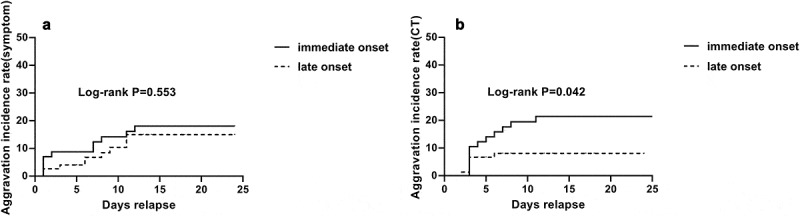
Incidence rate of aggravation for the two groups. (a) aggravation of symptoms (b) aggravation of CT presentation. There was no difference in symptom aggravation between the two groups (P = 0.553), but CT severity score elevated significantly in I group than in L group (P = 0.042)

We then analyzed the factors that influence disease aggravation in two aspects, aggravation of symptoms (presented by clinical types and ICU admission) and aggravation of CT presentation (presented by CT scores). Figure 2 shows the incidence curves of the two groups. The aggravation incidence of symptom was 13.3% in I group and 15.8% in L group, but there was no difference between the two groups (P = 0.553). The aggravation incidence of CT presentation was 21.1% in I group, significantly higher than L group (8.0%, P = 0.042). Results of COX analysis using different models are shown in Table 5. Adjusted by age and gender, incubation period, clinical types, treatment measures like glucocorticoids and immunoglobulin use, levels of lymphocytes counts, C reactive protein (CRP), interferon 6 (IL6) and symptoms like fatigue and headache were factors that influence CT aggravation. Multivariable COX models including different factors were also analyzed. The results suggested that incubation period was the only independent factors for CT aggravation in all the three models. The HRs were 0.40(95% CI: 0.19–0.84, P = 0.016), 0.36(95% CI: 0.14–0.94, P = 0.037) and 0.36(95% CI: 0.14–0.93, P = 0.035).

**Table 5.** Prognostic factors for CT aggravation by COX analysis

Factors	Adjusted COX analysis			Model 1		Model 2		Model 3		
	HR(95%CI)	P value	HR(95%CI)		P value	HR(95%CI)	P value	HR(95%CI)		P value
Onset of exposure to symptom	0.39(0.19–0.82)	0.012	0.40(0.19–0.84)		0.016	0.36(0.14–0.94)	0.037	0.36(0.14–0.93)	0.035	
Clinical type	2.12(1.06–4.25)	0.035	1.42(0.73–2.75)		0.307	1.08(0.37–3.16)	0.894	1.12(0.37–3.39)	0.840	
Glucocorticoids use	3.50(1.79–6.82)	<0.001	2.08(0.26–16.51)		0.489	2.07(0.23–18.57)	0.515	2.31(0.26–20.98)	0.456	
Immunoglobulin use	3.72(1.89–7.31)	<0.001	1.58(0.20–12.52)		0.666	0.77(0.08–7.44)	0.824	0.72(0.07–7.10)	0.782	
Lymphocytes	0.63(0.38–1.05)	0.075				0.82(0.39–1.71)	0.600	0.89(0.42–1.91)	0.770	
C reactive protein	2.67(1.30–5.48)	0.007				1.34(0.51–3.55)	0.557	1.34(0.50–3.59)	0.555	
IL6	3.21(1.21–8.54)	0.020				1.88(0.61–5.79)	0.269	1.86(0.62–5.59)	0.271	
Fatigue	2.86(1.43–5.70)	0.003						1.79(0.71–4.55)	0.219	
Headache	2.44(0.95–6.27)	0.064						1.05(0.23–4.78)	0.955	

**Model 1**: Age, Gender, Onset of exposure to symptom, Clinical type, Glucocorticoids use, Immunoglobulin use

**Model 2**: Age, Gender, Onset of exposure to symptom, Clinical type, Glucocorticoids use, Immunoglobulin use, Lymphocytes, C reactive protein, IL6

**Model 3**: Age, Gender, Onset of exposure to symptom, Clinical type, Glucocorticoids use, Immunoglobulin use, Lymphocytes, C reactive protein, IL6, Fatigue, Headache

## Discussion

Since the global spread of SARS-CoV-2 has become a huge threat to human being all over the world, scientists had made great efforts to reveal the epidemiological, clinical, and virological characteristics of SARS-CoV-2. Nevertheless, most of these studies focus on the clinical symptoms, treatment or prognosis of COVID-19; some studies also revealed the immunological mechanisms of disease deterioration [15]. However, few studies took onset time after exposure into consideration. During this time, patients, who were infected with SARS-CoV-2 with no symptoms, were thought to be contagious, which put a great challenge to public health [7,16]. A thorough understanding of the incubation period of infectious patients was the key to draw up an effective precaution measure [17]. Previous experiences from SARS indicated that rapidly determining the incubation period enabled public-health officials to set proper quarantine periods and terminate the transmission without the aid of treatment [18]. For COVID-19 this time, the satisfying results of diseases control in China also suggested the success of quarantine regulation [19]. More importantly, incubation period is the interaction of pathogen and host immunity. The length of incubation period is closely linked to immunological state [20], and the immunological state in part determined disease prognosis, presented clinically as asymptomatic carriers, disease aggravation, or recovery [21]. Consequently, a better understanding on the disease progression, especially evidenced by early symptoms, would help to optimize the current therapeutic strategies.

Some retrospective studies from confirmed COVID-19 cases have revealed certain factors that associated with disease progression. Study by Wei Hou et al., enrolling 101 diagnosed COVID-19 cases in Hubei, demonstrated that older age, increased CRP levels and decreased lymphocyte count were potential risk factors for disease progression [22]. In their study, the definition of the progression group was defined as either one of the three: increased clinical types, patients admitted to ICU or death during hospitalization. This definition was just consistent with one of our aggravation definition. But the difference lied in that our study proved that onset time of symptom had no significant influence on clinical aggravation. Apart from symptoms and body temperature, another study by Yulong Zhou et al. included chest CT presentation as one of the criteria for disease progression [21]. Their study suggested that higher total lymphocytes count was closely related to a better outcome of disease, which is also similar to our results. But after adjusted to other factors in multivariable models, total lymphocytes seemed not to be the independent factors for disease progression. Besides, some studies gave more specific result, indicating that CD4 T cell count was the independent factor for ICU admission [23]. Previous studies also showed that COVID-19 patients were susceptible to secondary infections due to complex immune dysfunction [24], and coinfection with bacteria could be seen in 30–50% severe or critically ill COVID-19 patients [25,26]. In our study, germiculture was not performed at baseline time, but antibiotics use (elevated serum WBC, CRP, and PCT levels), which might reflect bacterial infection to some aspect, was not an independent risk factor for disease progression.

Incubation period seemed to be the independent factors for disease aggravation in our study, with special reference to CT scores. However, several uncertainty affected the precise measurement of incubation period, of which the time of infection, usually bounding the time of exposure, was the most difficult to be determined. Thanks to the wide-spread of smartphone in China, the activity tracks of the infected could be traced with the aid of big data and artificial intelligence [27], providing a relatively correct exposure point. Previous studies had made some estimation of the incubation time of COVID-19. On the basis of known travel history to and from Wuhan, earlier studies from confirmed cases outside Wuhan suggested a mean incubation period of 5–6 days with a range of 2 to 14 days [28]. This is in line with the analysis of a familial cluster of COVID-19 after exposure [29]. These results indicated a similar incubation period of SARS-CoV-2 with SARS (mean, 5 days; range, 2 to 14 days) [2], MERS (mean, 5 to 7 days; range, 2 to 14 days) [3], and other human coronavirus (mean, 3 days; range, 2 to 5 days) [30]. A pooled analysis enrolling confirmed patients from 24 countries proved that the median incubation period was estimated to be 5.1 days (95% CI, 4.5 to 5.8 days), and 97.5% will develop symptoms within 11.5 days of infection [17]. The median incubation time in our study was 7.0 days, and17.3% patients start to have symptoms in less than 3 days after exposure, while 22.7% patients in more than 10 days. It seems that incubation period in our study was longer than these studies. Since more than 90% in our study were mild patients, we reckoned that the more severe the disease is, the shorter the incubation periods are.

The different incubation periods might be the presentation of different types of inflammation and immune responses. It has been proven that like other coronavirus infection, different types of immune responses involved in SARS-CoV-2 infection, including both innate and adaptive immune responses [31]. But differently, serum levels of cytotoxic T lymphocyte function-specific N proteins decrease in recovered patients, but are still detectable in peripheral blood mononuclear cell (PBMCs) from SARS or MERS patients 10 years post infection [32,33]. Actually, inflammatory reactions resembled what is observed in hypersensitivity pneumonitis rather than in other viral pneumonia, as proposed by Young et al [34]., therefore, three variants could be detected in SARS-CoV-2-related pneumonitis: acute, subacute and chronic. The final onset of symptom and pathophysiology is the results of genetics, environment, and immune reactions. Our study revealed that patients with different incubation periods had different prognosis, and the differences were mainly reflected by radiological characteristics. We presume that the difference of immune reaction lead to the different CT presentations. Actually, some has proposed that COVID-19 was the results of type III hypersensitivity reaction [35]. There was no difference in the aggravation of clinical symptoms or death, and we thought the reason was that most patients in our study were the mild patients.

Radiology presentations could be evaluated in many aspects, and CT scores were applied in our study. CT scores has been proven to be correlated with clinical and laboratory parameters in patients of pneumonia, first introduced in SARS cases [36], later were widely used in the research of COVID-19 radiology [14]. This semi-quantitative parameter provides a general damage degree, but could not tell the specific opacities involved. Different types of opacity like GGO, air trapping, parenchymal consolidation, et al. are associated with different immunological and pathological process. Paul J. Maglione et al. found that bronchiectasis was more strongly associated with infection and T-cell lymphopenia [37]. Our study revealed that GGOs in patients with shorter incubation period were less than patients of longer incubation period, but patients with shorter incubation period were more likely to get cavitation. The immunological mechanisms behind were worthy studying.

This study has several limitations. First, as a retrospective study, the detailed symptom change could not be obtained, so we took the aggravation of clinical type as the criteria in combination with the radiological characteristics. A more detailed therapeutic responses in a cohort study should be designed. Second, there is a lack of a predictive model for disease progression to evaluate our conclusions. Third, although big data provide a relatively correct exposure time, the reality is complicated to achieve a precise incubation time. Fourth, time for CT reexamination was not fixed, so it was likely that the period between two CT examinations in severe patients was shorter than mild patients. Fifth, a thorough classification of immune cells and immunological experiment should be analyzed to compare the difference between the two groups. Finally, since patients with longer incubation time were less likely to aggravate, further studies should include asymptomatic carriers into analysis

In summary, our study demonstrated that patients with different incubation periods were different in clinical symptoms, laboratory tests, and CT presentations. Shorter incubation was associated with the aggravation of lung involvement in CT scan. Further treatment should focus more attention on patients with shorter incubation period.

## Data Availability

The data that support the findings of this study are available from the corresponding author upon reasonable request.
